# Perceived Social Support Partially Mediates the Impact of Temperament and Character on Postpartum Depression

**DOI:** 10.3389/fpsyt.2021.816342

**Published:** 2022-01-24

**Authors:** Yukako Nakamura, Nagahide Takahashi, Aya Yamauchi, Mako Morikawa, Takashi Okada, Norio Ozaki

**Affiliations:** ^1^Department of Psychiatry, Nagoya University Graduate School of Medicine, Nagoya, Japan; ^2^Department of Child and Adolescent Psychiatry, Nagoya University Graduate School of Medicine, Nagoya, Japan; ^3^Psychiatry/Child and Adolescent Psychiatry, Nagoya University Hospital, Nagoya, Japan; ^4^Department of Developmental Disorders, National Center of Neurology and Psychiatry, Kodaira, Japan

**Keywords:** cohort study, depression, postpartum, personality, perceived social support, mediation analysis

## Abstract

**Introduction:**

Temperament and character of pregnant women, especially harm avoidance (HA) and self-directedness (SD) have been identified as risk factors for postpartum depression, in addition to poor social support. However, the relationship between these personality traits and social support for depressive symptoms after delivery has not been examined.

**Methods:**

Data were extracted from a prospective cohort survey on pregnant women conducted in Nagoya, Japan that included the Temperament and Character Inventory (TCI), the Social Support Questionnaire (J-SSQ), and the Edinburgh Postnatal Depression Scale (EPDS) at approximately week 25 and 1 month postpartum. A mediation analysis using structural equation modeling (SEM) was used to test if social support in pregnancy is a mediator between personality traits and postpartum depressive symptoms.

**Results:**

Thousand five hundred and fifty-nine women were included in the analysis. Both harm avoidance and SD were significantly associated with depressive symptoms (total effect: β [SE], 0.298 [0.041], *P* < 0.001 for harm avoidance; total effect: β [SE], −0.265 [0.067], *P* < 0.001 for SD). Mediation analysis showed that the effect of harm avoidance on depressive symptoms was partially mediated by low social support (direct effect: β [SE], 0.193 [0.004], *P* < 0.001; indirect effect: β [SE], 0.082 [0.034], *P* = 0.015). Self-directedness on depressive symptoms was not found to be mediated by low social support.

**Conclusion:**

Results indicate that poor social support worsens depressive symptoms in women with high HA during pregnancy. Limitations include a possible selection bias due to the limited target facilities; most variables being evaluated based on self-report questionnaires, and different number of samples available for analysis between harm avoidance and SD.

## Introduction

Postpartum depression, which has an approximate prevalence of 10–15% in mothers (1–3), can lead to maternal suicide (4–6) and the developmental failure of the child (7–9). Thus, identification of the risk for postpartum depression, and protective factors against its development, is critical (10, 11). It has been pointed out that the involvement of temperament and personality in the development of depression using various questionnaires or inventories (12–14). Among them, research between depression and temperament and character (13, 15) using the Temperament and Character Inventory (TCI) developed by Cloninger et al. (16) has been extensively studied (13–18) and a longitudinal study has demonstrated that TCI is clinically useful for predicting future risk of developing depression (19).

Temperament and Character Inventory is based on a seven-dimensional model consisting of temperament (four dimensions) and personality (three dimensions). The four dimensions of temperament are Novelty Seeking (NS), Harm Avoidance (HA), Reward Dependence (RD), and persistence (P), and the three dimensions of personality are, self-directedness (SD), Cooperativeness (C), and Self-Transcendence (ST) (20). Of the seven dimensions proposed by Cloninger et al., HA and SD have been repeatedly reported to be particularly associated with depression (13, 17, 18, 21, 22). Cloninger et al. proposed Temperament being stable, with a biological basis, and Character being acquired (16, 20). However, subsequent research confirmed the state-dependence of temperament (23, 24), and recently the distinction between the constructs of temperament and personality has been called into question (25).

Previously we found that scores of HA increase with the severity of depressive symptoms (24), and that HA predicts the development of postpartum depression (26). Earlier studies of perinatal women pointed to an association between HA and SD with depression during pregnancy (27, 28), as well as postpartum depression, using relatively small samples (29). It has also been reported that personality, measured 2–3 days after delivery, predicts depression at 8 and 32 weeks after delivery (30). Taken together, it is possible that assessment of personality of perinatal women is important as it may identify pregnant women at risk for development of postpartum depression. However, there have been no studies that have evaluated the association of personality traits in pregnancy with the risk of postpartum depression using a large sample size.

In addition to the above, social support during pregnancy has been shown to be protective against postpartum depression (31). However, it has not been elucidated whether social support is protective in women with high-risk personality traits for postpartum depression. Using structural equation modeling (SEM), Kendler et al. examined factors involved in depression for women, and also examined social support (32), but they evaluated social support in late adolescence, not in the perinatal period. In order to examine risk factors and protective factors for perinatal depression, it is necessary to evaluate personality traits as well as social support in the perinatal period.

In the present study, therefore, we used SEM to clarify the relationship between personality traits, social support, and postpartum depression using data from our prospective perinatal cohort and identify protective factors in subjects with high-risk personality traits for the development of postpartum depression.

## Materials and Methods

### Design

Data from this study were extracted from a prospective cohort study carried out in Nagoya, Japan, from August 2004 to March 2020. Three questionnaires, the Edinburgh Postnatal Depression Scale (EPDS), TCI, and Japanese version of Social Support Questionnaire (J-SSQ) were used to measure depressive symptoms and related factors. The socio-demographic variables included age, partner's age, and number of children. Participants answered the questionnaire during early pregnancy (T1: around 25 weeks of gestation) which included the EPDS, TCI, and J-SSQ. One month after delivery (Q2), the EPDS, were filled out and the questionnaire was mailed back. This study followed the STrengthening the Reporting of OBservational studies in Epidemiology (STROBE) reporting guideline.

### Participants

Participants were recruited from a general hospital, two obstetric and gynecological hospitals and a university hospital. Pregnant women participating in prenatal classes before the 25th week of pregnancy were invited to participate if they were aged 20 years or older and able to read and write Japanese. Since the age of adulthood in Japan is 20 years, we excluded pregnant women under the aged 20 years in this study.

### Ethical Considerations

A written informed consent was obtained from all those who agreed to participate in the study. The study protocol was approved by the Ethics Committee of the Nagoya University Graduate School of Medicine and all study procedures met the Committee's guidelines and regulations. All study protocols were in accordance with the 1964 Helsinki Declaration and its later amendments.

### Measurements

#### TCI

Personality traits including HA and SD were measured using the TCI. The TCI is a self-report questionnaire consisted of 125 items that look at four dimensions of temperament (novelty seeking, HA, reward dependence, and persistence) and three dimensions of character (SD, cooperativeness, and self-transcendence). We used the Japanese version of the TCI-125, which includes 125 questions covering 20 items pertaining to HA (33). The reliability and validity of the Japanese version of TCI-125 have been previously confirmed (34, 35). Harm avoidance scores ranged from 0 to 20 and SD scores ranged from 0 to 25. Harm avoidance and SD in the early pregnancy (around week 25) were used for the analysis. We started assessing the HA in August 2004, and SD in April 2011.

#### J-SSQ

We used the J-SSQ (36) to measure perceived social support among pregnant women in the present study. The J-SSQ has two factors: “Number of Persons” and “Satisfaction Rating” (31). “Number of Person” reflects the average number of persons the participants perceive to be available to provide social support to them. “Satisfaction Rating” reflects an individual's average degree of satisfaction for the perceived support to be available (31). The reliability and validity of J-SSQ has been confirmed in the pregnancy and postpartum periods (36). The J-SSQ in the early pregnancy (around week 25) was used for the analysis.

#### EPDS

The EPDS is a self-administered questionnaire developed by Cox et al. for the screening of postpartum depression. EPDS comprises 10 items and was scored on a four-point scales with total scores ranging from 0 to 30 (37). The Japanese version of the EPDS by Okano et al. (38) was used in this study and scores above nine were utilized for the screening of major depressive episodes (sensitivity, 82%; specificity, 95%) (39, 40). Participants' depressive symptoms were evaluated using the EPDS 1 month after birth.

### Statistical Analyses

The statistical analyses were performed in Stata, version 16. To test if social support is a mediator between personality traits and postpartum depressive symptoms, we performed SEM analysis with maximum likelihood. Missing values were estimated by full information maximum likelihood method (FIML). The analysis of the outcome (total EPDS scores) included all observed and latent variables in a single step. The *medsem* command in Stata was used to estimate indirect effects in the mediation model (41). We used the Monte Carlo method to test the significance of the indirect effects as described (42). Comparative fit index (CFI) and root mean squared error of the approximation (RMSEA) were used to evaluate a goodness of fit for each model.

## Results

### Demographics

In total, 1,559 pregnant women were included in the analysis who submitted valid questionnaires at around the 25th week of pregnancy. The average age of the participants was 32.4 ± 4.6 years (*n* = 1,542). There were 1,138 (72.9%) primiparas, 381 (24.4%) multiparas, and 40 unanswered about parity.

### Correlation Between Variables

**Figure 1:**
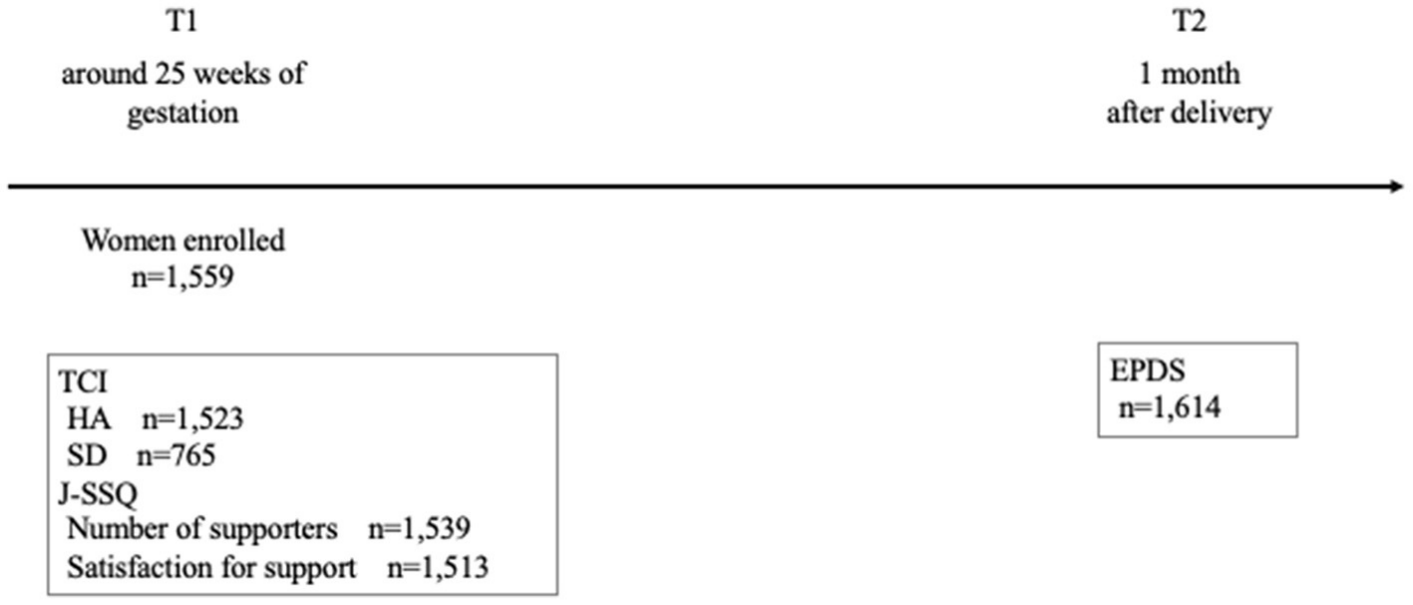
Procedure for recruitment of subjects. TCI, Temperament and Character Inventory; J-SSQ, Japanese version of the Social Support Questionnaire; EPDS, Edinburgh Postnatal Depression Scale.

Table 1 shows the summary of all the variables and Table 2 shows the correlations between the variables. The EPDS score at 1 month after delivery was positively correlated with HA (*r* = 0.259, *p* < 0.01) and negatively correlated with SD (*r* = −0.344, *p* < 0.01), J-SSQ Number (*r* = −0.111, *p* < 0.01), and Satisfaction (*r* = −0.097, *p* < 0.01). The recruitment process was described in Figure 1.

**Table 1:** Summary of measured variables.

	***n***	**Means**	**Standard deviation**	**Skewness**
Age	1,542	32.4	4.6	0.04
T1 (TCI) harm avoidance	1,523	11.6	4.5	−0.32
T1 (TCI) self-directedness	765	17.9	4.7	−0.95
T1 (J-SSQ) number of supporters	1,539	3.9	2.2	3.60
T1 (J-SSQ) satisfaction with support	1,513	4.9	1.2	−1.92
T2 EPDS total score	1,416	5.1	4.7	1.27

*T1, Time point 1(around 25 weeks of pregnancy); T2, Time point 2 (1 month after delivery); TCI, Temperament and Character Inventory; J-SSQ, Japanese version of the Social Support Questionnaire; EPDS, Edinburgh Postnatal Depression Scale*.

**Table 2:** Correlations between variables.

	**T1 HA**	**T1 SD**	**T1 NS**	**T1 SS**	**T2 EPDS**
T1 HA_TCI	1.000				
T1 SD_TCI	−0.543**	1.000			
T1 NS_J-SSQ	−0.226**	0.262**	1.000		
T1 SS_J-SSQ	−0.139**	0.203**	0.154**	1.000	
T2 EPDS	0.259**	−0.344**	−0.111**	−0.097**	1.000

*T1, Time point 1(around 25 weeks of gestation); T2, time point 2 (1 month after delivery); HA, Harm Avoidance; SD Self-Directedness; TCI, Temperament and Character Inventory; NS, Number of supporters; SS, Satisfaction for support; J-SSQ, Japanese version of the Social Support Questionnaire; EPDS, Edinburgh Postnatal Depression Scale*.

** *p < 0.01*.

### Mediation Analysis

**Figure 2:**
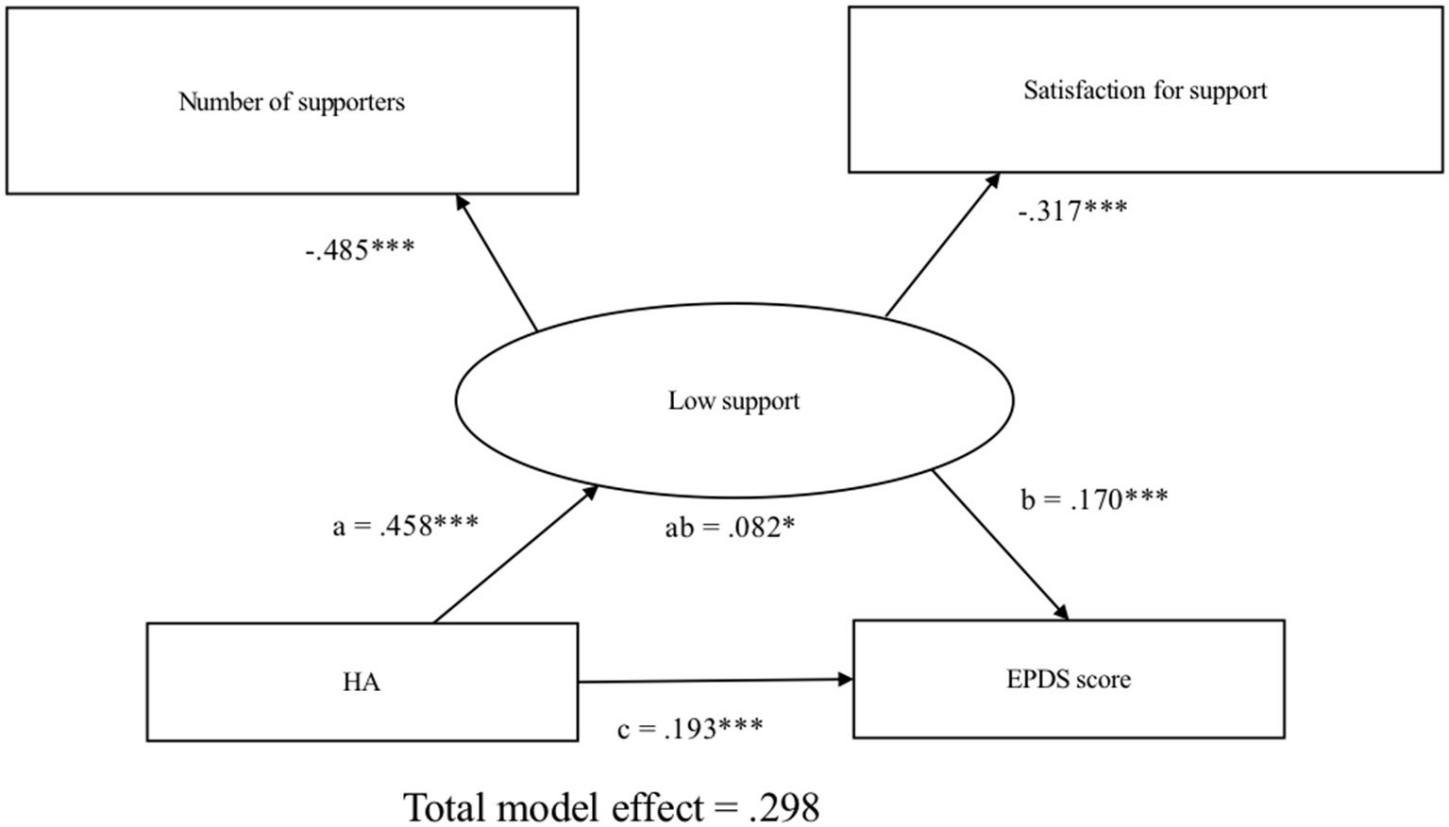
Structural equation modeling of harm avoidance, social support, and postpartum depressive symptoms. Observed measures are represented by squares and latent variables are represented by circles. Single-headed arrows (path) define causal relationships between variables. Numbers represents standardized coefficient. a, effect of HA on low support; b, effect of low support on EPDS total scores; c, direct effect of HA on EPDS total scores; ab, indirect effect of HA on EPDS total scores. HA, harm avoidance; EPDS, Edinburgh Postnatal Depression Scale. *P < 0.05, **P < 0.01, ***P < 0.001.

Figure 2 illustrates the model that shows the association between HA and EPDS scores (CFI = 1.00, RMSEA < 0.001). Harm avoidance is significantly associated with EPDS scores (total effect: β [SE], 0.298 [0.041], *P* < 0.001). Mediation analysis showed that the effect of HA on depressive symptoms was partially mediated by low social support (direct effect: β [SE], 0.193 [0.004], *P* < 0.001; indirect effect: β [SE], 0.082 [0.034], *P* = 0.015), indicating that 29.8% of the total effect of HA on EPDS scores was mediated through low social support.

**Figure 3:**
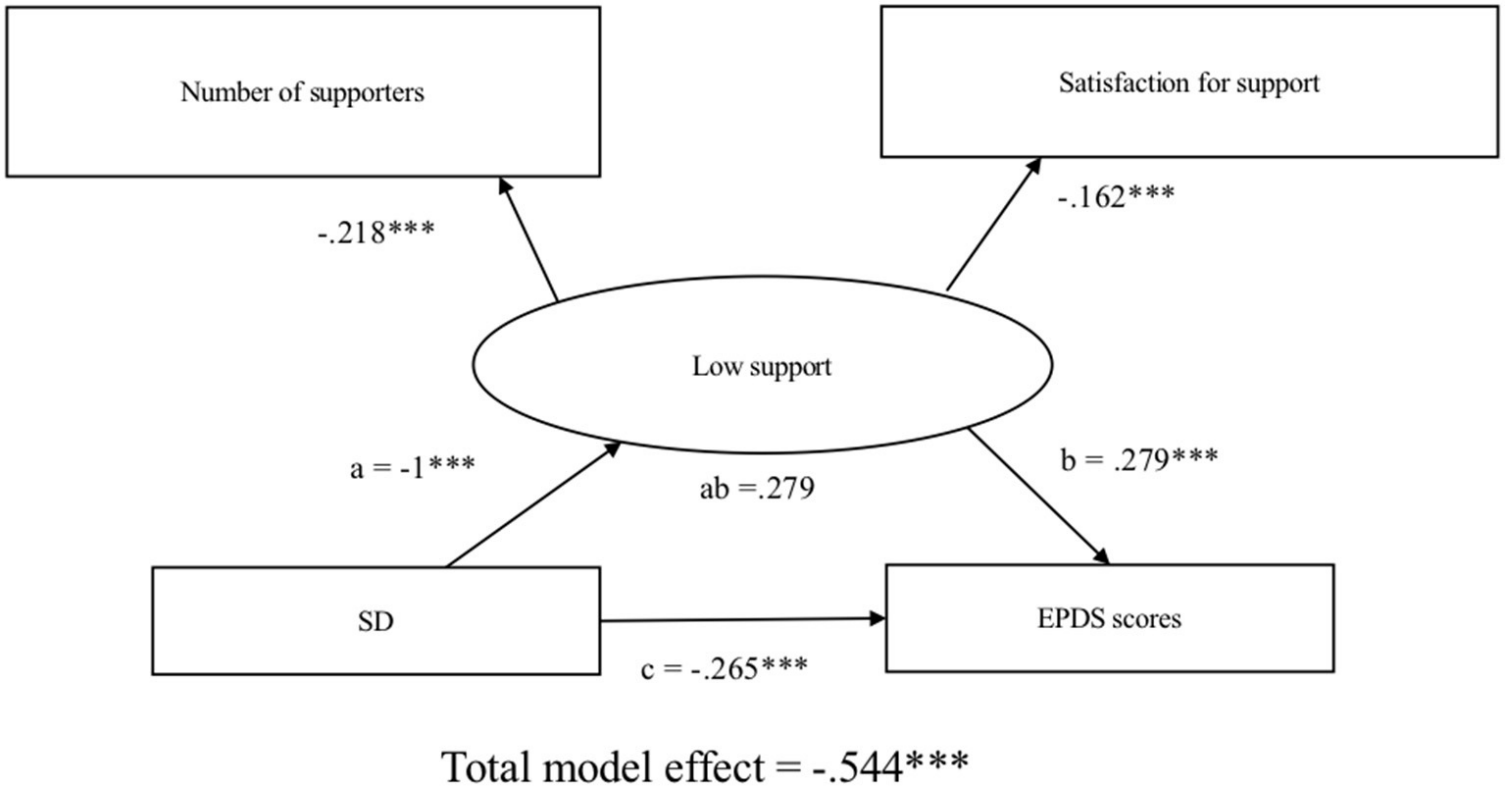
Structural equation modeling of self-directedness, social support, and postpartum depressive symptoms. Observed measures are represented by squares and latent variables are represented by circles. Single-headed arrows (path) define causal relationships between variables. Numbers represents standardized coefficient. a, effect of SD on low support; b, effect of low support on EPDS total scores; c, direct effect of SD on EPDS total scores; ab, indirect effect of SD on EPDS total scores. SD, self-directedness; EPDS, Edinburgh Postnatal Depression Scale. *P < 0.05, **P < 0.01, ***P < 0.001.

Figure 3 illustrates the model that shows the association between SD and EPDS scores (CFI = 1.00, RMSEA < 0.001). Self-directedness is significantly associated with EPDS scores (total effect: β [SE], −0.544 [0.067], *P* < 0.001). Mediation analysis showed that the effect of SD on depressive symptoms was not mediated by low social support (direct effect: β [SE], −0.265 [0.248], *P* < 0.001; indirect effect: β [SE], 0.279 [0.000], *P* = 0.094).

## Discussion

In the present study, we confirmed that personality traits during pregnancy predict the depressive symptomatology at 1 month postpartum. Additionally, we identified social support during pregnancy as a mediator for the severity of depressive symptoms in women at high risk for postpartum depression, which could be a potential target for intervention to protect the development of the illness.

Our results showed that high HA and low SD around 25 weeks' gestation are associated with higher EPDS scores in the first month of postpartum, which successfully replicates previous studies of postpartum depression using relatively small sample sizes (27–29). In one of the previous results of our cohort study [Kubota et al. (26)], we reported that high HA during pregnancy predicted postpartum depression. However, HA during pregnancy did not predict postpartum depression in Furumura et al. (24). One of the possible reasons for this discrepancy is that the number of samples was small in Furumura's report, where only 99 participants responded for HA items. In this study, we expanded the sample size and confirmed that both HA and SD during pregnancy predict postpartum depression.

We have also confirmed that social support during pregnancy is a protective factor for postpartum depression. This result was consistent with our previous papers (31, 43–45). The mediation analysis showed that social support could be a partial mediator for postpartum depressive symptoms in high HA subjects, but not in low SD subjects. One possible explanation is the use of different coping strategies between high HA and low SD subjects, which is supported by a recent study using office workers (46). This study suggests low HA is associated with problem-focused coping skills, whereas low SD is associated with emotion-focused coping skills. Therefore, it could be considered that subjects with high HA had difficulty in coping with their stressors when using a problem-focused approach, which may have led to not seeking social support or underestimating the actual social support available to them. On the other hand, HA has been reported to be associated with anxiety symptoms (47) and we have previously shown that EPDS has three-factor consisting of anxiety, depression, and anhedonia (48). Future study which examines social support specifically affect these specific factors of EPDS is needed. In contrast, high SD subjects had difficulty in utilizing emotion-focused coping skills (46), and thus their depressive symptoms might be independent of the availability of social support. Intensive psychological care, such as cognitive behavioral therapy, might be effective for those subjects, rather than an increase in social support (49, 50).

Some limitations should be considered in this study. First, there is a possible selection bias due to the limited target facilities. In addition, we included only pregnant women who participated in the perinatal class. Second, the variables used in this study were mostly evaluated based on self-report questionnaires, not on a diagnosis by a psychiatrist nor a structured clinical interview. Third, we started evaluating HA, J-SSQ, and EPDS in 2004, while evaluating SD started in 2011. Therefore, the number of samples available for analysis differs between HA and SD, which may have affected the results. Finally, there is a possibility that HA and SD itself has been affected by maternal symptoms.

In conclusion, in this study we have shown that social support may work differently with specific personality traits on depressive symptoms after delivery. It is important to provide appropriate support according to mothers' temperament and characteristics to prevent the development of postpartum depression.

## Data Availability Statement

The raw data supporting the conclusions of this article will be made available by the authors, without undue reservation.

## Ethics Statement

The studies involving human participants were reviewed and approved by the Ethics Committee of the Nagoya University Graduate School of Medicine. The patients/participants provided their written informed consent to participate in this study.

## Author Contributions

YN, NT, AY, and NO conceptualized, designed the study, and interpreted the data. YN, NT, AY, TO, MM, and NO acquired and analyzed the data. YN, NT, and NO drafted the manuscript, tables, and figures. All authors approved the final version to be published.

## Funding

This work was supported by research grants from the Ministry of Education, Culture, Sports, Science and Technology of Japan; the Ministry of Health, Labor and Welfare of Japan; the Academic Frontier Project for Private Universities, Comparative Cognitive Science Institutes, Meijo University; the Core Research for Evolutional Science and Technology; Intramural Research Grant (21B-2) for Neurological and Psychiatric Disorders from the National Center of Neurology and Psychiatry and the New Technology Development Foundation (the Specific Research Fund 2012 for East Japan Great Earthquake Revival to TO); Research and Development Grants for Comprehensive Research for Persons with Disabilities from the Japan Agency for Medical Research and Development (AMED, JP20dk0307077, JP21wm0425007, JP21dm0207075, JP21dk0307103 to NO); JSPS KAKENHI (JP20K07943 to YN and 21K07479 to NT).

## Conflict of Interest

The authors declare that the research was conducted in the absence of any commercial or financial relationships that could be construed as a potential conflict of interest.

## Publisher's Note

All claims expressed in this article are solely those of the authors and do not necessarily represent those of their affiliated organizations, or those of the publisher, the editors and the reviewers. Any product that may be evaluated in this article, or claim that may be made by its manufacturer, is not guaranteed or endorsed by the publisher.
